# Night Shift Work, Chronotype, Sleep Duration, and Prostate Cancer Risk: CAPLIFE Study

**DOI:** 10.3390/ijerph17176300

**Published:** 2020-08-29

**Authors:** Macarena Lozano-Lorca, Rocío Olmedo-Requena, María-Victoria Vega-Galindo, Fernando Vázquez-Alonso, Antonio Jiménez-Pacheco, Inmaculada Salcedo-Bellido, María-José Sánchez, José-Juan Jiménez-Moleón

**Affiliations:** 1 Universidad de Granada, Departamento de Medicina Preventiva y Salud Pública, 18016 Granada, Spain; macarenalozano@ugr.es (M.L.-L.); isalcedo@ugr.es (I.S.-B.); mariajose.sanchez.easp@juntadeandalucia.es (M.-J.S.); jjmoleon@ugr.es (J.-J.J.-M.); 2 Instituto de Investigación Biosanitaria (ibs.GRANADA), 18014 Granada, Spain; 3 Consortium for Biomedical Research in Epidemiology and Public Health (CIBERESP), 28029 Madrid, Spain; 4 Góngora Primary Health Care Centre, Distrito Sanitario Granada-Metropolitano, 18006 Granada, Spain; maveggal@gmail.com; 5 Urology Department, Virgen de las Nieves University Hospital, 18014 Granada, Spain; fvazquezalonso@gmail.com; 6 Urology Department, San Cecilio University Hospital, 18016 Granada, Spain; anjipa29@hotmail.com; 7 Andalusian School of Public Health (EASP), 18011 Granada, Spain

**Keywords:** prostatic neoplasms, sleep, shift work schedule, circadian rhythm, case-control studies, CAPLIFE study

## Abstract

To analyze the association between prostate cancer (PCa) risk and night shift work, chronotype, and sleep duration in the context of a population-based case-control study of incident prostate cancer in Spain, a total of 465 PCa cases and 410 controls were analyzed. Selection criteria were: (i) age 40–80 years, and (ii) residence in the coverage area of the reference hospitals for ≥6 months before recruitment. Exposure variables were: (i) night shift work (permanent or rotating); (ii) chronotype: morning, neither, or evening (Munich ChronoType Questionnaire) and (iii) sleep duration according to the recommendations of the American National Sleep Foundation. PCa aggressiveness was determined according to the International Society of Urology Pathology classification. Adjusted odds ratios (aOR) and 95% confidence intervals (95% CI) were estimated using logistic regression models. Night shift work was associated with PCa, aOR = 1.47 (95% CI 1.02–2.11), especially for rotating night shifts, aOR = 1.73 (95% CI 1.09–2.75). The magnitude of the association between ever night work and PCa was higher in evening subjects with aOR = 3.14 (95% CI 0.91–10.76) than in morning chronotypes with an aOR = 1.25 (95% CI 0.78–2.00). Working night shifts, especially rotating night shifts, could increase PCa risk. This risk may be higher in people with an evening chronotype.

## 1. Introduction

Prostate cancer (PCa) is the most frequent cancer in males and has the third-highest mortality in Europe [1]. PCa incidence has increased in all countries in recent years, although with high variations geographically [2,3]. In Spain, it is estimated that 35,126 new cases of PCa are diagnosed, according to the latest estimates from the Spanish Cancer Registry Network, this being the most frequent cancer in men [4]. By 2040, more than 2 million men are estimated to be affected [1]. However, despite its considerable impact, the etiology of PCa is still unknown. Established risk factors include older age, race/ethnicity, and family history, all nonmodifiable risk factors [5,6,7,8]. At present, the list of carcinogenic agents classified by the International Agency for Research on Cancer (IARC, Lyon, France) does not include any with sufficient evidence in humans for PCa [9]. There is limited evidence for some agents such as cadmium or arsenic, consumption of red meat, or occupational exposures such as night shift work, but the results of epidemiological studies are inconsistent and unclear [10,11,12,13,14].

In Europe, approximately 21% are night shift workers, and 23% in Spain [15]. According to data from the U.S. Bureau of Economic Analysis, this percentage ranges from 9.1% to 13.9%, depending on the definition of night shift in the U.S. [16]. Night shift work involves night tasks, and it could cause a mismatch between a person’s circadian phase and their work cycle schedule, known as “circadian disruption” [17]. Chronic disruption of the circadian rhythm is associated with a variety of health risks including metabolic diseases and cancer [18]. In addition, chronic disruption of the circadian rhythm promotes tumor proliferation and inhibition of apoptosis [19]. Biologically, this higher incidence and proliferation of the tumor among night workers could be caused by exposure to light at night, which entails the suppression of the production of melatonin, a hormone whose production takes place in the absence of light and has oncostatic properties [20].

Accordingly, in 2019, the IARC Working Group reevaluated the association between night shift work and PCa [21], which was previously evaluated in 2007 [22], confirming its classification in Group 2A: “probably carcinogenic to humans.” As the authors themselves comment, this classification was based on sufficient evidence from experimental animal models but limited evidence for human cancer, especially for breast, prostate, and colorectal cancer [21]. In addition, this group identified several major domains of night shifts and shift schedules that should be captured in future studies: shift system (permanent or rotating), years on a night shift schedule, and shift intensity [23]. Since its classification as a possible carcinogen in 2007, several epidemiological studies have evaluated the association between night shift work and PCa risk, although their results do not always point in the same direction [13,24,25,26,27,28,29]. Hence, IARC, in the latest report in 2019, noted considerable variability in details on the night shifts reported in these studies and concluded that studies were few, and the results lacked consistency [21,30].

Shift work may modify sleep habits and chronotype [31,32]. Along these lines, an association between sleep duration and cancer has been described, finding a relationship between a deficit of hours of sleep and a higher risk of breast and colorectal cancer [33,34]. This risk association could be due to the existence of reduced levels of melatonin, as it suppresses the initiation phase of tumorigenesis and inhibits the proliferation of human cancer cell lines [35]. However, for PCa, no association has been observed previously [27,36]. Chronotype has been traditionally conceptualized as an individual preference to sleep at a particular time; however, at present, it is known that it is determined by internal factors (e.g., clock genes, cortisol, and melatonin levels) and environmental factors (e.g., social habits, light/dark cycle, and season), differentiating between “morning”, “intermediate or neither”, and “evening” chronotype [37,38,39]. The latter has been associated with a higher risk of breast cancer [40]; however, PCa the results are scarce and a definitive association is unknown [27]. In this sense, the possible modifying effect of the chronotype on the relationship between shift work and cancer has been recently suggested [41]. Therefore, discrepancies between studies analyzing the association between night shift work and PCa may also be due to the lack of consideration of the role that chronotype plays in this possible association, and only a few studies have considered chronotype due to its uncertain role [13,25,27,29]. It has been proposed that a better alignment of chronotype with rotating or night shift work may lead to less circadian disruption, and hence influence the association between PCa and night shift work [42].

Given the increase in PCa incidence trend, the high percentage of night shift workers, together with the scarcity and inconsistency of studies that explore this association, as recognized by the IARC Working Group, and also the scarce consideration of the role of chronotype in this possible association, we consider it necessary to carry out this study. Additionally, PCa cannot be a unique pathology, and the association of night shift work with PCa may depend on its aggressiveness and individual characteristics [13,28,29]. Hence, we aimed to analyze the association between PCa risk and night shift work, chronotype, and sleep duration, in the context of a population-based case-control study of incident prostate cancer out in Spain (CAPLIFE study).

## 2. Materials and Methods

### 2.1. Study Design and Setting

The design of the CAPLIFE study has been previously described [43]. Briefly, this study is a population-based case-control study that was developed to investigate the association between lifestyles and PCa risk. It was carried out at the two main University Hospitals in Granada (Spain)—Virgen de las Nieves and San Cecilio Hospitals—and their catchment areas. This study was approved by the Ethics Committee of Biomedical Research of Andalusia in March 2017. All subjects were fully informed about the study objectives and signed an informed consent form.

### 2.2. Participants

Eligible cases met the following selection criteria: (1) new diagnosis of PCa with histological confirmation (International Classification of Diseases 10th Revision [ICD-10]: C61 [44]) before starting any type of treatment for PCa including active surveillance; (2) age between 40 and 80 years; and (3) residence in the coverage area of the reference hospitals for at least six months before recruitment. Controls were selected using the same selection criteria except for the diagnosis of PCa. Cases were recruited and invited to participate in the urology services of both hospitals. Controls were chosen at random from the general population using the lists of general practitioners at primary health centers. They were frequency-matched by age (±5 years) based on data regarding the frequency of PCa cases by age obtained from the population-based Granada Cancer Registry. Both PCa patients and participants of the control group were invited to participate from May 2017 to March 2020.

### 2.3. Data Collection

Face-to-face interviews were conducted by trained clinical researchers. Information on the following variables was obtained: socio-demographic data; lifestyle including smoking status, alcohol drinking, and physical activity; body mass index (BMI); occupational history; and personal/family medical history including first-grade family history of PCa. Information on physical activity was collected through the International Physical Activity Questionnaire Short Form (IPAQ-SF), validated for the Spanish population, which establishes the existence of three levels of physical activity: low, moderate, and high physical activity [45]. Regarding sleep and night shift work, the following information was collected: night shift work (permanent or rotating shift work), chronotype, and sleep duration. In the case of missing information, participants were contacted by telephone to complete the missing information.

Clinical information of PCa cases were extracted from medical records including Gleason score and stage at diagnosis.

### 2.4. Night Shift Work

Occupational history data were obtained for all jobs that lasted at least a year including company, location, tasks involved, start and stop dates, and shift work. Ever night shift work was defined as working partly or entirely (≥3 h) between 22:00 and 06:00, at least three times per month; as it is legally considered in Spain (Statute of Workers’ Rights) [46]. A distinction was made between (i) permanent night work, defined as one that only includes night shifts, without having morning or afternoon shifts; and (ii) rotating shift work, defined as any rotation between morning, evening, and/or night shifts. If a participant reported both, its duration was considered and they were included in the category that had a longer duration. The reference group consisted of men who had never performed night shift work for at least a year. Rotating shift workers with no night shifts were included in the reference group (never night shift).

Of those subjects with ever night shift, the following night shift indicators were calculated: (i) lifetime cumulative duration as the total number of years worked at night, regardless of the number of shift nights; and (ii) intensity, as the number of shift nights per year. Terciles calculated with the control group and the cut-offs points were applied to the cases.

### 2.5. Chronotype

Chronotype was evaluated using the Munich ChronoType Questionnaire (MCTQ) at 40 years old [47]. This was estimated, using the methodology described in previous studies, as the mid-sleep time on free days [MSF = (sleep onset on free day + sleep duration on free day)/2)], corrected for oversleep on free days compared to working days [MSFsc = MSF − (sleep duration on free day − sleep duration on a working day)/2] [47,48]. This tool has been previously used in the Spanish population [13,49,50,51]. Chronotype were categorized as: (i) morning type: MSFsc <04:00 a.m.; (ii) neither type: MSFsc ≥4:00–≤5:00 a.m.; and (iii) evening type: MSFsc >05:00 a.m.

### 2.6. Sleeps Duration

In the same way, sleep duration was assessed by the MCTQ at 40 years old [47]. To calculate this, we subtracted sleep onset from sleep end. The subjects were classified according to their adherence to the recommendations of the American National Sleep Foundation (Seattle, WA, USA): (i) recommended sleep duration (≥7:00–≤9:00 a.m.); (ii) may be appropriated (≥6:00–<7:00 a.m. or >9:00–≤10:00 a.m.); and (iii) not recommended (<6:00 h or >10:00 a.m.) [52].

### 2.7. Measurement of Tumor Aggressiveness

The Gleason score was collected from the pathology report. The aggressiveness of the tumor was determined according to the International Society of Urological Pathology classification (ISUP, USA), which establishes five grades: (i) ISUP 1 (Gleason 3 + 3); (ii) ISUP 2 (Gleason 3 + 4); (iii) ISUP 3 (Gleason 4 + 3); (iv) ISUP 4 (Gleason 8); and (v) ISUP 5 (Gleason >8) [53]. From this, two categories of aggressiveness were constructed: low aggressiveness (ISUP 1–2) and high aggressiveness (ISUP 3–5) [43,54].

### 2.8. Statistical Analysis

In the descriptive analysis, the mean and standard deviation (SD) of the continuous quantitative variables and percentages for categorical variables were calculated for the comparison of characteristics between PCa cases and controls and across case and control groups according to the type of work. Chi-squared tests were used to evaluate the level of significance of the differences observed in categorical variables, and the Student´s and one-way ANOVA tests for continuous variables.

Multivariable logistic regression models were used to estimate the odds ratios (OR) and 95% confidence intervals (95% CI) for the association between PCa risk and shift night work, chronotype, and sleep duration. Models included the following adjustment variables: age, education, first-grade family history of PCa, physical activity, and smoking status. The reference group consisted of subjects who had never performed night shifts for at least one year. We evaluated chronotype as a possible effect modifier for the association between night shift work including night-shift indicators and PCa risk. In addition, we carried out the analysis of the association between the variables of interest and the PCa stratified by aggressiveness.

All statistical tests were two-sided and statistical significance was set at *p* < 0.05. Statistical analyses were performed using statistical program Stata v.15 (Stata Corp., 2017, College Station, TX, USA).

## 3. Results

**Figure 1:**
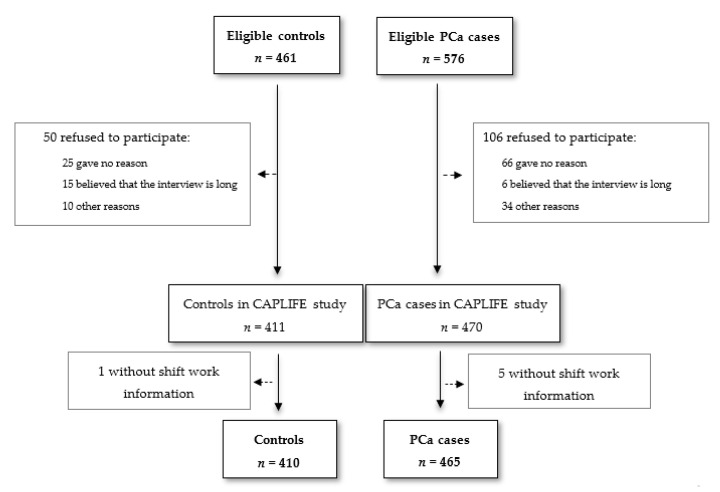
Flow-chart CAPLIFE study.

Figure 1 shows the flowchart diagram for the participants in the CAPLIFE study. A total of 465 PCa cases (98.9% of all PCa cases that met the selection criteria) and 410 controls (99.8% of the total number of controls that met the selection criteria) had complete information about shift work. Distribution of the characteristics of controls and PCa cases, differentiated by type of work (never/ever night shift), are shown in Table 1. Compared to the controls, PCa cases were slightly older, 67.7 years (SD 7.5) vs. 65.6 (SD 7.9). No differences were found for the level of education, BMI, smoking status, physical activity, and first-degree family history of PCa. More than half of the PCa cases were of very low aggressiveness (58.7%), with an ISUP 1. While among the controls, it was those who ever worked at night who had a higher frequency of university studies (31.8% for ever night workers vs. 19.2% for never night workers in the control group), among the cases, the frequency was inverse for university studies (10.3% for cases ever night workers vs. 19.3% for controls never night workers). More information on the distribution of the characteristics according to the type of work can be found in Supplementary Table 1 (Table S1).

Table 2 shows the associations between PCa risk and night shift work, chronotype, and sleep duration. The percentage of ever night workers was higher among PCa cases (20.9%) than the controls (16.1%). We observed a risk association between ever night work and PCa with an adjusted Odds Ratio (aOR) = 1.47 (95% CI 1.02–2.11). The participants with rotating night shifts were those who presented a higher risk of PCa, aOR = 1.73 (95% CI 1.09–2.75). However, no association was found between the type of chronotype or sleep duration and risk of PCa: aOR = 1.18 (95% CI 0.72–1.93) for evening vs. morning chronotype, and aOR = 1.18 (95% CI 0.80–1.74) for participants with no recommended sleep duration compared to those who adhered to sleep recommendations. The aggressiveness of PCa does not appear to modify the association between night shift work and PCa risk (Supplementary Table 2, Table S2).

The associations between the number of years accumulated as well as the intensity of night work and PCa risk are shown in Table 3. There was no clear trend between a longer duration of night work and the risk of PCa. The shift intensity was associated with a greater risk of PCa in rotating shift workers with an aOR = 2.60 (95% CI 1.34–5.02) for those who had at least 74 night shifts per year.

In the analysis stratified by chronotype, a possible interaction with the type of shift work was observed, although it did not reach statistical significance (Table 4). The aOR of having ever performed night shift was highest among subjects with an evening chronotype of aOR = 3.14 (95% CI 0.91–10.76) compared to subjects with morning chronotype of aOR = 1.25 (95% CI 0.78–2.00). The highest risks were for evening subjects with permanent night or rotating night shift work with an aOR = 3.53 (95% CI 0.76–16.34) and aOR = 2.72 (95% CI 0.54–13.62), respectively.

## 4. Discussion

In brief, the results of our study indicate a risk association between night shift work and PCa, and specifically for rotating night shift work. Our results also point toward a possible interaction between evening chronotype and night shift work, increasing the PCa risk. The aggressiveness of PCa does not seem to modify the association between night shift work and PCa risk.

As the IARC points out in its latest report on shift work and cancer, few studies have evaluated night shift work and PCa risk and these showed inconsistent results [21]. The IARC emphasized the need for captured information on the shift work system (permanent or rotating), years on a non-day shift schedule, and shift intensity [23,30]. We have considered their recommendations, differentiating between permanent and rotating night shifts, calculating the total number of years working a night shift, and the intensity of night shifts.

Concerning work shifts, we observed that it could increase the risk of PCa. Our results are in line with Tse et al. [24], where a case-control study was carried out in a Chinese population, and Behrens et al. [25], who undertook a German population-based cohort study. In the same way, the MCC-Spain study suggests a higher risk of PCa of aOR = 1.14 (95% CI 0.94–1.37) for subjects who had ever worked night shifts for at least one year [13]. However, these results contrast with the absence of association in other studies such as the EPICAP (Epidemiological study of prostate cancer), a French case-control study, and PROtEuS (Prostate Cancer and Environment Study), a case-control study conducted in Canada, and Swedish and Finnish cohorts [26,27,28,29]. One of the main issues that could justify this inconsistency between studies is the varying definition of night shift work used. No set single definition exists, and occupational information collected on the night shift differs between studies. In this sense, we have used the definition of shift work established by the relevant authorities on labor matters in Spain and collected information on all work carried out throughout life [46]. Other studies have considered all one-year jobs, but did not establish a minimum number of hours or night shifts per month to be determined as night work, as in the case of the Swedish cohort [26] or only the current or latest work, as in the Finnish cohort [27]. In the same way, EPICAP included all 6-month-long jobs and used the French legal definition of night work (to work at least 270 h at night per year or three nights per month) [29] and the PROtEuS study defined night-shift work as having ever worked for at least 3 h between midnight and 5:00 a.m. for at least one year with a minimum frequency of three nights per month [28]. This last definition is not exactly the same as ours as it includes a shorter night time period. According to our results, it was those who worked on rotating night shifts who presented the greatest risk; this same trend was observed by the MCC-Spain study [13]. Even though the IARC Working Group contemplates the existence of two types of night work, permanent, and rotating night work [21], this distinction was not used in the Swedish cohort nor the Chinese case-control [24,26]. It should be noted that we only found a risk trend for rotating night work but not for permanent night work. A possible explanation could be the occurrence of greater chronodisruption in rotating night shift workers. Along these lines, a recent study revealed that rotating night shift workers have later and less pronounced melatonin peaks than day shift workers [42]. A similar pattern has been found for other types of tumors such as colorectal cancer [51]. However, more studies are necessary to confirm the grade of chronodisruption depending on the type of shift work.

In addition, we did not find a clear trend between exposure time in night work and PCa risk, in line with previous studies [26,28,29]. The MCC-Spain study and a German cohort found a greater risk trend as years of exposure increased [13,25], however, the results are not comparable when using different cut-off points. Most studies use cut-off points established from terciles or quartiles, depending on the study population [13,28], and this makes the comparison of results difficult. We must also take into account other indicators of night shifts such as the time since the cessation of night shift work. Thus, although a recent study indicates that the risk of PCa for subjects with night shift work could be null after 20 years of cessation of night work [55], we did not observe this association (data not shown). However, we would have to consider the lack of statistical power in our study to address this last objective.

We observed a possible interaction between the type of individual chronotype and night shift work. As previously mentioned, it has been proposed that a better alignment of chronotype with rotating or night shift work may lead to less circadian disruption, and hence influence the association between PCa and night shift work [42]. Furthermore, evening types have been thought to adapt better to night shifts; however, evening types have also been shown to have poorer sleep quality for both day and night shifts [56]. This may explain our findings of an increased risk in those with an evening chronotype. Previous studies have also evaluated this, and two studies found that evening men with night shift work could be more at risk for PCa than morning types with a similar job in night shift, further supporting our results [27,29]. However, in the German cohort, it was suggested that it is the morning ones with a night shift that would present a higher risk than the evening ones with a night shift [25], while the MCC-Spain study found no interaction [13]. These possible discrepancies may be due to the use of different definitions of work shifts, as previously mentioned, along with the application of different instruments to measure the chronotype. So, although in the German cohort study the midpoint of the night on free days was used to calculate the chronotype, they used the 25th and 75th percentiles to establish the cut-off point between morning–intermediate and intermediate–night chronotypes, respectively [25]. In contrast, we used the MCTQ, a questionnaire widely used to determine the chronotype [32,57,58] including in the Spanish population [13,49,50,51], with cut-off points established a priori.

We have not found an association between an inadequate number of hours of sleep and the risk of PCa. This is in line with three previous cohort studies [27,36,59], even when different categorizations of sleep duration are used, differentiating between sleeping adequately, sleeping excessively, and the sleeping default. It should be borne in mind that measuring the duration of sleep is not easy, especially when referring back in time. Furthermore, perhaps not only the duration of sleep can be related to PCa, but also the quality of sleep, an aspect not addressed in our study, and which would be interesting to address in further research. Some limitations may have been able to affect the results found. Among them, the sample size limitation should be noted, especially in analyses stratified by chronotype and aggressiveness and the evaluation of the interaction between chronotype and night shift work, where numbers are smaller, and we might have lacked statistical power to detect significant associations and interactions. Second, we must highlight the difficulty in collecting information on the main exposure variables, given the variability over time of work type, its schedules, and sleep duration. Recall bias cannot be totally ruled out, although it has been minimized by the use of standardized questionnaires and similar interviewing conditions for cases and controls. We attempted to overcome this by collecting information on all jobs carried out, defining night work according to the Statute of Workers’ Rights, and measuring the chronotype on days off and using the MCTQ [47]. Information on lifetime occupational history and on work time schedule was self-reported by the cases and controls, which may have induced a classification bias. However, if it were to occur, it would be non-differential and attenuate risk estimates toward the null. In addition, given the absence of recommended cut-off points, we used terciles to measure the years and intensity of night shifts, which are dependent on the study population and make comparability between studies difficult. Although we adjusted for a range of potential confounders, which were associated with both night shift work and PCa risk, we cannot rule out the absence of confounding by other occupational exposures/agents related to night shift work and cancer.

Our study also has several advantages, and the following should be highlighted: (i) the use of a widely used questionnaire for collecting data on sleep duration and chronotype at 40 years [47] as the age of 40 has been used in previous studies to measure sleep duration [13,50,51]; (ii) we collected information on all jobs with at least one year in duration, and not just the last job, increasing the external validity of the results; (iii) we followed the recommendations of the IARC Working Group, distinguishing between rotating and permanent night shift work and calculating the cumulative lifetime duration, and intensity of night work [21,23]; (iv) most PCa cases (98.9%) and controls (99.8%) had work shift information; and (v) although there could be a memory bias, it would affect both groups, and therefore it would be non-differential, underestimating the results obtained. Finally, the probable carcinogenicity of night shift work is still not a major concern in Spain, and men were informed about the objectives of the CAPLIFE study—to analyze the association between lifestyles and PCa—but not specifically the effects of night shift work.

## 5. Conclusions

To conclude, in this Spanish population-based case-control study, an association was observed between night shift work and PCa risk, specifically for rotating night shift work. This association was similar in low and high aggressive PCa. In addition, a higher risk can be observed in night shift workers with an evening chronotype. Determining the etiology of PCa is not easy, in part because of the biological heterogeneity of the disease. Therefore, prevention of PCa is challenging given that established risk factors including age, race/ethnicity, family history, and genetic variants are primarily nonmodifiable. We should consider that the use of results from observational studies to establish causal relationships is limited due to multiple complicating and intractable issues. Therefore, more studies are necessary to confirm this relationship including a good definition of exposure, working with incident PCa cases, and considering the individual chronotype as a possible effect modifier.

## Figures and Tables

**Table 1:** Characteristics controls and PCa cases according to having been ever/never night work. CAPLIFE study.

	Controls			PCa Cases			
	Never Night Shift N = 344	Ever Night Shift N = 66	Total N = 410	Never Night Shift N=368	Ever Night Shift N = 97	Total N = 465	*P*-Value ^a^
**Age (Years), Mean (SD)**	66.5 (7.7)	63.2 (8.4)	65.6 (7.9)	67.4 (7.5)	68.4 (7.6)	67.7 (7.5)	**0.001**
***P-*** **Value ^b^**	**0.002**				0.244		
**Age (Years), N (%)**							**0.039**
40–54	28 (8.1)	14 (21.2)	42 (10.2)	24 (6.5)	4 (4.1)	28 (6.0)	
55–69	182 (52.9)	41 (62.1)	223 (54.4)	198 (53.8)	50 (51.6)	248 (53.3)	
70–80	134 (39.0)	11 (16.7)	145 (35.4)	146 (39.7)	43 (44.3)	189 (40.7)	
***P-*** **Value ^b^**	**<0.001**			0.544			
**Education, N (%)**							0.357
Primary	100 (29.1)	19 (28.8)	119 (29.0)	108 (29.3)	36 (37.1)	144 (31.0)	
Secondary	178 (51.7)	26 (39.4)	204 (49.8)	189 (51.4)	51 (52.6)	240 (51.6)	
University	66 (19.2)	21 (31.8)	87 (21.2)	71 (19.3)	10 (10.3)	81 (17.4)	
***P-*** **Value ^b^**	0.053			0.079			
**BMI, Mean (SD)**	28.4 (3.9)	28.9 (4.6)	28.5 (4.0)	28.2 (4.1)	28.0 (3.6)	28.2 (4.0)	0.252
***P-*** **Value ^b^**	0.400			0.598			
**BMI, N (%)**							0.569
Normal weight (<25 Kg/m^2^)	65 (18.9)	8 (12.1)	73 (17.8)	75 (20.4)	21 (21.6)	96 (20.6)	
Overweight (25–29.9 Kg/m^2^)	179 (52.0)	38 (57.6)	217 (52.9)	184 (50.0)	54 (55.7)	238 (51.2)	
Obesity (≥30 Kg/m^2^)	100 (29.1)	20 (30.3)	120 (29.3)	109 (29.6)	22 (22.7)	131 (28.2)	
***P-*** **Value ^b^**	0.411			0.396			
**Smoking Status, N (%)**							0.781
Never	89 (25.9)	19 (28.8)	108 (26.3)	95 (25.8)	23 (23.7)	118 (25.4)	
Former	189 (54.9)	36 (54.5)	225 (54.9)	195 (53.0)	56 (57.7)	251 (54.0)	
Current	66 (19.2)	11 (16.7)	77 (18.8)	78 (21.2)	18 (18.6)	96 (20.6)	
***P-*** **Value ^b^**	0.832			0.700			
**Physical Activity (MET-Hour/Week), Mean (SD)**	29.4 (29.7)	35.4 (49.6)	30.3 (33.7)	27.9 (32.6)	32.3 (30.9)	28.8 (32.3)	0.489
***P-*** **Value ^b^**	0.186			0.234			
**Physical Activity, N (%)**							0.171
Low	119 (34.6)	19 (28.8)	138 (33.7)	152 (41.3)	32 (33.0)	184 (39.6)	
Moderate	177 (51.4)	36 (54.5)	213 (52.0)	176 (47.8)	49 (50.5)	225 (48.4)	
High	48 (14.0)	11 (16.7)	59 (14.3)	40 (10.9)	16 (16.5)	56 (12.0)	
***P-*** **Value ^b^**	0.625			0.176			
**First-Degree Family History of PCa ^c^, N (%)**							0.771
No	324 (94.2)	64 (97.0)	388 (94.6)	348 (94.6)	89 (91.8)	437 (94.0)	
Yes	20 (5.8)	2 (3.0)	22 (5.4)	19 (5.1)	8 (8.2)	27 (5.8)	
Unknown				1 (0.3)		1 (0.2)	
***P-*** **Value ^b^**	0.358			0.251			
Aggressiveness *, N (%)							
ISUP 1				207 (56.3)	66 (68.0)	273 (58.7)	
ISUP 2				71 (19.3)	13 (13.4)	84 (18.1)	
ISUP 3				30 (8.2)	7 (7.2)	37 (8.0)	
ISUP 4				41 (11.1)	6 (6.2)	47 (10.1)	
ISUP 5				18 (4.9)	5 (5.2)	23 (4.9)	
***P-*** **Value ^b^**				0.263			

BMI, Body Mass Index; PCa, Prostate cancer; SD, standard deviation; **^a^** Student’s t-tests or Chi-squared test were used to calculate the differences between PCa cases and controls. **^b^** Student’s t-tests or Chi-squared test were used to calculate the differences between types of work. **^c^** First-degree history of PCa in father and/or brothers. * One subject could not be categorized using ISUP classification, as it was a neuroendocrine carcinoma. Values with a *p*-value ≤0.050 are highlighted in bold.

**Table 2:** Associations between PCa and night shift work, chronotype, and sleep duration.

	Controls N = 410	PCa Cases N = 465	*P*-Value ^a^	aOR ^b^ (95% CI)
	*n* (%)	*n* (%)		
**Shift Work**				
Never night shift	344 (83.9)	368 (79.1)	0.071	1
Ever night shift	66 (16.1)	97 (20.9)		1.47 (1.02–2.11)
**Types of Night** **Shift**				
Never night shift			0.073	1
Permanent night shift	34 (8.3)	39 (8.4)		1.21 (0.73–1.98)
Rotating night shift	32 (7.8)	58 (12.5)		1.73 (1.09–2.75)
**Chronotype ^c^**				
Morning	247 (60.2)	283 (60.8)	0.622	1
Neither	125 (30.5)	130 (28.0)		0.94 (0.69–1.28)
Evening	34 (8.3)	45 (9.7)		1.18 (0.72–1.93)
Missing	4 (1.0)	7 (1.5)		
**Sleep Duration ^c^**				
Recommended	217 (52.9)	234 (50.3)	0.784	1
May be appropriated	124 (30.2)	146 (31.4)		1.11 (0.81–1.50)
Not recommended	65 (15.9)	78 (16.8)		1.18 (0.80–1.74)
Missing	4 (1.0)	7 (1.5)		

**^a^** Unadjusted p-values calculated using Chi-squared tests. **^b^** Adjusted for age, education, first-degree family history of PCa, physical activity, and smoking status. **^c^** Assessed with Munich ChronoType Questionnaire (MCTQ) at 40 years.

**Table 3:** Associations between night work indicators and PCa risk according to the type of night shift work.

	Ever Night Shift			Permanent Night Shift			Rotating Night Shift		
	Controls N = 66	PCa Cases N = 97	aOR ^a^ (95% CI)	Controls N = 34	PCa Cases N = 39	aOR ^a^ (95% CI)	Controls N = 32	PCa Cases N = 58	aOR ^a^ (95% CI)
	N (%)	N (%)		N (%)	N (%)		N (%)	N (%)	
**Lifetime Cumulative Duration of Night Shifts (Years)**									
Never night shift			1			1			1
Tercile 1: ≤7	23 (5.6)	29 (6.2)	1.33 (0.75–2.38)	18 (4.3)	16 (3.4)	0.97 (0.48–1.95)	5 (1.2)	13 (2.8)	2.57 (0.90–7.34)
Tercile 2: >7–≤26	22 (5.4)	40 (8.6)	1.93 (1.11–3.36)	8 (2.0)	11 (2.4)	1.58 (0.61–4.06)	14 (3.4)	29 (6.2)	2.08 (1.08–4.04)
Tercile 3: >26	21 (5.1)	28 (6.0)	1.18 (0.65–2.14)	8 (2.0)	12 (2.6)	1.37 (0.54–3.47)	13 (3.2)	16 (3.5)	1.08 (0.51–2.29)
**Intensity of Night Shift** **(Night Shifts/Year)**									
Never night shift			1			1			1
Tercile 1: ≤74	22 (5.4)	20 (4.3)	0.88 (0.46–1.65)	4 (1.0)			18 (4.4)	20 (4.3)	1.05 (0.54–2.03)
Tercile 2: >74–≤250	23 (5.6)	47 (10.1)	2.06 (1.21–3.49)	10 (2.4)	12 (2.6)	1.26 (0.52–3.05)	13 (3.2)	35 (7.5)	2.60 (1.34–5.02)
Tercile 3: >250	20 (4.9)	27 (5.9)	1.40 (0.76–2.58)	20 (4.9)	27 (5.8)	1.40 (0.76–2.58)			
Unknown ^b,^*	1 (0.2)	3 (0.6)					1 (0.2)	3 (0.6)	

**^a^** Adjusted for age, education, first-degree family history of PCa, physical activity, and smoking status. ^**b**^ It is not possible to calculate the number of nights shifts, because these subjects had irregular rotating shifts without a fixed number of nights per month. * aOR with less than 10 cases and controls were not calculated.

**Table 4:** Associations between night work indicators and PCa risk according to chronotype.

	Morning Chronotype			Neither Chronotype			Evening Chronotype			
	Controls N = 247	PCa Cases N = 283	aOR ^a^ (95% CI)	Controls N = 125	PCa Cases N = 130	aOR ^a^ (95% CI)	Controls N = 34	PCa Cases N = 45	aOR ^a^ (95% CI)	*P*-Interaction
**Shift Work**										
Never night shift	208 (84.2)	232 (82.0)	1	109 (87.2)	106 (81.5)	1	27 (79.4)	29 (64.4)	1	0.280
Ever night shift	39 (15.8)	51 (18.0)	1.25 (0.78–2.00)	16 (12.8)	24 (18.5)	1.71 (0.83–3.51)	7 (20.6)	16 (35.6)	3.14 (0.91–10.76)	
**Types of Night Shift**										
Never night shift			1			1			1	0.199
Permanent night shift	20 (8.1)	19 (6.7)	0.99 (0.50–1.94)	9 (7.2)	11 (8.5)	1.64 (0.61–4.40)	4 (11.8)	9 (20.0)	3.53 (0.76–16.34)	
Rotating night shift	19 (7.7)	32 (11.3)	1.50 (0.82–2.75)	7 (5.6)	13 (10.0)	1.77 (0.66–4.73)	3 (8.8)	7 (15.6)	2.72 (0.54–13.62)	
**Lifetime Cumulative Duration of Night Shift (Years)**										
Never night shift			1			1			1	0.107
Tercile 1: ≤7	11 (4.5)	15 (5.3)	1.40 (0.62–3.19)	9 (7.2)	11 (8.5)	1.56 (0.58–4.21)	3 (8.8)	3 (6.7)	1.26 (0.19–8.47)	
Tercile 2: >7–≤26	12 (4.8)	22 (7.7)	1.78 (0.85–3.76)	4 (3.2)	9 (6.9)	2.51 (0.72–8.75)	4 (11.8)	7 (15.6)	2.58 (0.54–12.37)	
Tercile 3: >26	16 (6.5)	14 (5.0)	0.78 (0.37–1.66)	3 (2.4)	5 (3.1)	1.14 (0.24–5.39)		6 (13.3)		
**Intensity of Night** **Shift** **(Nights Shifts/Year)**										
Never night work			1			1			1	0.450
Tercile 1: ≤74	16 (6.5)	11 (3.9)	0.61 (0.27–1.37)	4 (3.2)	5 (3.9)	1.17 (0.29–4.66)	1 (3.0)	3 (6.7)	3.54 (0.26–48.22)	
Tercile 2: >74–≤250	12 (4.8)	28 (9.9)	2.22 (1.09–4.55)	6 (4.8)	11 (8.5)	2.13 (0.72–6.30)	3 (8.8)	6 (13.3)	2.87 (0.55–14.84)	
Tercile 3: >250	11 (4.5)	12 (4.2)	1.14 (0.48–2.71)	6 (4.8)	8 (6.2)	1.69 (0.52–5.49)	3 (8.8)	7 (15.6)	3.29 (0.62–17.50)	

^a^ Adjusted for age, education, first-degree family history of PCa, physical activity and smoking status.

## Supplementary Materials

The following are available online at https://www.mdpi.com/1660-4601/17/17/6300/s1, Table S1: Characteristics controls and PCa cases according to night shift frequency (never, rotating, permanent), Table S2: Associations between PCa risk and type of night shift work, chronotype, and sleep duration stratified by aggressiveness.
