# Supplementary material for: Night Shift Work, Chronotype, Sleep Duration, and Prostate Cancer Risk: CAPLIFE Study
Source: Int J Environ Res Public Health. 2020 Aug 29;17(17):6300. doi: 10.3390/ijerph17176300 (PMC7503878; doi:10.3390/ijerph17176300)
Supplement: Supplementary file 1 [file ijerph-17-06300-s001.pdf]

Supplementary Table S1. Characteristics controls and PCa cases according to night shift frequency (never, roatating, permanent).

	Controls			PCa Cases		
	**Never night shift n=344**	**Permanent night shift n=34**	**Rotating night shift n=32**	**Never night shift n=368**	**Permanent night shift n=39**	**Rotating night shift n=58**
**Age (years), mean (SD)**	66.5 (7.7)	61.1 (8.6)	65.4 (7.5)	67.4 (7.5)	68.1 (7.6)	68.7 (7.6)
***p*-value[a]**		**<0.001**			0.471	
**Age (years), n (%)**						
40–54	28 (8.1)	10 (29.4)	4 (12.5)	24 (6.5)	-	4(6.9)
55–69	182 (52.9)	20 (58.8)	21 (65.6)	198 (53.8)	22 (56.4)	28 (48.3)
70–80	134 (39.0)	4 (11.8)	7 (21.9)	146 (39.7)	17 (43.6)	26 (44.8)
***p*-value[a]**		**<0.001**			0.544	
**Education, n (%)**						
Primary	100 (29.1)	8 (23.6)	11 (34.4)	108 (29.3)	36 (37.1)	144 (31.0)
Secondary	178 (51.7)	13 (38.2)	13 (40.6)	189 (51.4)	51 (52.6)	240 (51.6)
University	66 (19.2)	13 (38.2)	8 (25.0)	71 (19.3)	10 (10.3)	81 (17.4)
***p*-value[a]**		0.095			0.500	
**BMI, mean (SD)**	28.4 (3.9)	28.4 (4.9)	29.4 (4.2)	28.2 (4.1)	27.3 (3.7)	28.4 (3.4)
***p*-value[a]**		0.411			0.377	
**BMI, n (%)**						
Normal weight (<25 Kg/m^2)	65 (18.9)	6 (17.6)	2 (6.2)	75 (20.4)	11 (28.2)	10 (17.2)
Overweight (25 – 29.9 Kg/m^2)	179 (52.0)	19 (55.9)	19 (59.4)	184 (50.0)	21 (53.8)	33 (56.9)
Obesity (≥30 Kg/m^2)	100 (29.1)	9 (26.5)	11 (34.4)	109 (29.6)	7 (18.0)	15 (25.9)
***p*-value[a]**		0.498			0.438	
**Smoking status, n (%)**						
Never	89 (25.9)	12 (35.3)	7 (21.9)	95 (25.8)	7 (18.0)	16 (27.6)
Former	189 (54.9)	16 (47.1)	20 (62.5)	195 (53.0)	22 (56.4)	34 (58.6)
Current	66 (19.2)	6 (17.6)	5 (15.6)	78 (21.2)	11 (25.6)	8 (13.8)
***p*-value[a]**		0.691			0.529	
**Physical activity (MET-hour/week), mean (SD)**	29.4 (29.7)	43.8 (65.7)	26.4 (20.1)	27.9 (32.6)	31.4 (36.9)	32.9 (26.5)
***p*-value[a]**		**0.045**			0.481	
**Physical activity, n (%)**						
Low	119 (34.6)	7 (20.6)	12 (37.5)	152 (41.3)	15 (38.5)	17 (23.3)
Moderate	177 (51.4)	18 (52.9)	18 (56.3)	176 (47.8)	19 (48.7)	30 (51.7)
High	48 (14.0)	9 (26.5)	2 (6.2)	40 (10.9)	5 (12.8)	11 (19.0)
***p*-value[a]**		0.133			0.315	
**First-degree family history of PCa[b], n (%)**						
No	324 (94.2)	33 (97.1)	31 (96.9)	348 (94.6)	36 (92.3)	53 (91.4)
Yes	20 (5.8)	1 (2.9)	1 (3.1)	19 (5.1)	3 (7.7)	5 (8.6)
Unknown	–	–	–	1 (0.3)	–	–
***p*-value[a]**		0.655			0.508	
**Aggressiveness[*], n (%)**						
ISUP 1	–	–	–	207 (56.3)	26 (66.7)	40 (69.0)
ISUP 2	–	–	–	71 (19.3)	6 (15.4)	7(12.0)
ISUP 3	–	–	–	30 (8.2)	3 (7.7)	4 (6.9)
ISUP 4	–	–	–	41 (11.1)	2 (5.1)	4 (6.9)

ISUP 5	–	–	–	18 (4.9)	2 (5.1)	3 (5.2)
***p*-value**[a]		–			0.685	

BMI, Body Mass Index; PCa, Prostate cancer; SD, standard deviation; [a]One-way ANOVA or Chi-squared test were used to calculate the differences. [b]First-degree history of PCa in father and/or brothers. [*]One subject could not be categorized using ISUP classification, as it was a neuroendocrine carcinoma.

Supplementary Table S2. Associations between PCa risk and type of night shift work, chronotype, and sleep duration stratified by aggressiveness.

	Low aggressiveness PCa cases[a] n=357	aOR[b] (95% CI)	High aggressiveness PCa cases[a] n=107	aOR[b] (95% CI)
**Shift work**				
Never night shift	278 (77.9)	1	88 (83.2)	1
Ever night shift	79 (22.1)	**1.57 (1.09 – 2.28)**	18 (16.8)	1.29 (0.71 – 2.34)
**Types of night shift**				
Never night shift		1		1
Permanent night shift	32 (8.9)	1.29 (0.77 – 2.17)	7 (6.5)	1.07 (0.44 – 2.60)
Rotating night shift	47 (13.2)	**1.85 (1.15 – 3.00)**	11 (10.3)	1.48 (0.70 – 3.11)
**Lifetime cumulative duration of night shift (years)**				
Never night shift		1		1
Tercile 1: ≤ 7	27 (7.5)	1.60 (0.89 – 2.89)	2 (1.8)	0.44 (0.10 – 1.94)
Tercile 2: > 7 – ≤ 26	32 (9.0)	**1.96 (1.10 – 3.48)**	8 (7.5)	1.97 (0.81 – 4.77)
Tercile 3: > 26	20 (5.6)	1.16 (0.66 – 2.37)	8 (7.5)	1.50 (0.62 – 3.62)
**Intensity of night shift (nights shifts/year)**				
Never night work		1		1
Tercile 1: ≤ 74	17 (4.8)	0.99 (0.51 – 1.92)	3 (2.8)	0.59 (0.17 – 2.06)
Tercile 2: > 74 – ≤ 250	38 (10.6)	2.23 (1.28 – 3.87)	9 (8.4)	1.58 (0.68 – 3.67)
Tercile 3: > 250	22 (6.2)	1.43 (0.75 – 2.71)	5 (4.7)	1.62 (0.56 – 4.68)
Unknown[c, *]	2 (0.5)	–	1 (0.9)	–
**Chronotype**[d]				
Morning	214 (59.9)	1	68 (63.5)	1
Neither	102 (28.6)	0.96 (0.69 – 1.34)	28 (26.2)	0.87 (0.52 – 1.46)
Evening	35 (9.8)	1.18 (0.70 – 1.98)	10 (9.4)	1.24 (0.56 – 2.74)
Missing	6 (1.7)		1 (0.9)	
**Sleep duration**[d]				
Recommended	174 (48.7)	1	59 (55.1)	1
May be appropriated	110 (30.8)	1.12 (0.80 – 1.56)	36 (33.7)	1.02 (0.63 – 1.67)
Not recommended	67 (18.8)	1.34 (0.90 – 2.01)	11 (10.3)	0.73 (0.35 – 1.51)
Missing	6 (1.7)		1 (0.9)	

[a]Categories based on the recategorization of aggressiveness according to the classification of International Society of Urological Pathology (ISUP): low (ISUP 1 – 2) and high aggressiveness (ISUP 3 – 5). One subject could not be categorized using ISUP classification, as it was a neuroendocrine carcinoma.[b]Adjusted for age, education, first-degree family history of PCa, physical activity, and smoking status. [c]It is not possible to calculate the number of nights shifts, because these subjects had irregular rotating shifts, without a fixed number of nights per month. [*]aOR with less than 10 cases and controls were not calculated. [d]Assessed with Munich ChronoType Questionnaire (MCTQ) at 40 years.
